# Integrated experimental and technoeconomic evaluation of two-stage Cu-catalyzed alkaline–oxidative pretreatment of hybrid poplar

**DOI:** 10.1186/s13068-018-1124-x

**Published:** 2018-05-17

**Authors:** Aditya Bhalla, Peyman Fasahati, Chrislyn A. Particka, Aline E. Assad, Ryan J. Stoklosa, Namita Bansal, Rachel Semaan, Christopher M. Saffron, David B. Hodge, Eric L. Hegg

**Affiliations:** 1 DOE Great Lakes Bioenergy Research Center, Michigan State University, 1129 Farm Lane, East Lansing, MI 48824 USA; 2 Department of Biochemistry & Molecular Biology, Michigan State University, 603 Wilson Road, East Lansing, MI 48824 USA; 3 Department of Biosystems & Agricultural Engineering, Michigan State University, 216 Farrall Hall, East Lansing, MI 48824 USA; 4 Department of Chemical Engineering & Materials Science, Michigan State University, 428 S. Shaw Lane, East Lansing, MI 48824 USA; 5 Division of Sustainable Process Engineering, Luleå University of Technology, 98187 Luleå, Sweden; 6 Present Address: Faculdade de Engenharia Agrícola, UNICAMP, Cândido Rondon, 501, Cidade Universitária, Campinas, São Paulo 13083-875 Brasil; 7 Present Address: Department of Chemical and Biological Engineering, 3111 Engineering Hall, 1415 Engineering Drive, Madison, WI 53706 USA; 8 Present Address: Sustainable Biofuels and Co-Products Research Unit, Eastern Regional Research Center, USDA, ARS, 600 E. Mermaid Lane, Wyndmoor, PA 19038 USA; 9 Present Address: Chemical and Biological Engineering Department, Montana State University, PO Box 173920, Bozeman, MT 59717 USA

**Keywords:** Alkaline hydrogen peroxide (AHP), Biofuels, Copper, Hybrid poplar, Lignin, Lignocellulosic biomass, Oxidative delignification, Technoeconomic analysis (TEA)

## Abstract

**Background:**

When applied to recalcitrant lignocellulosic feedstocks, multi-stage pretreatments can provide more processing flexibility to optimize or balance process outcomes such as increasing delignification, preserving hemicellulose, and maximizing enzymatic hydrolysis yields. We previously reported that adding an alkaline pre-extraction step to a copper-catalyzed alkaline hydrogen peroxide (Cu-AHP) pretreatment process resulted in improved sugar yields, but the process still utilized relatively high chemical inputs (catalyst and H_2_O_2_) and enzyme loadings. We hypothesized that by increasing the temperature of the alkaline pre-extraction step in water or ethanol, we could reduce the inputs required during Cu-AHP pretreatment and enzymatic hydrolysis without significant loss in sugar yield. We also performed technoeconomic analysis to determine if ethanol or water was the more cost-effective solvent during alkaline pre-extraction and if the expense associated with increasing the temperature was economically justified.

**Results:**

After Cu-AHP pretreatment of 120 °C NaOH-H_2_O pre-extracted and 120 °C NaOH-EtOH pre-extracted biomass, approximately 1.4-fold more total lignin was solubilized (78% and 74%, respectively) compared to the 30 °C NaOH-H_2_O pre-extraction (55%) carried out in a previous study. Consequently, increasing the temperature of the alkaline pre-extraction step to 120 °C in both ethanol and water allowed us to decrease bipyridine and H_2_O_2_ during Cu-AHP and enzymes during hydrolysis with only a small reduction in sugar yields compared to 30 °C alkaline pre-extraction. Technoeconomic analysis indicated that 120 °C NaOH-H_2_O pre-extraction has the lowest installed ($246 million) and raw material ($175 million) costs compared to the other process configurations.

**Conclusions:**

We found that by increasing the temperature of the alkaline pre-extraction step, we could successfully lower the inputs for pretreatment and enzymatic hydrolysis. Based on sugar yields as well as capital, feedstock, and operating costs, 120 °C NaOH-H_2_O pre-extraction was superior to both 120 °C NaOH-EtOH and 30 °C NaOH-H_2_O pre-extraction.

**Electronic supplementary material:**

The online version of this article (10.1186/s13068-018-1124-x) contains supplementary material, which is available to authorized users.

## Background

Increasing energy demands, the desire for energy independence, and growing concern over greenhouse gas emissions and global warming have encouraged the search for renewable, eco-friendly sources of energy, including biofuels produced from lignocellulosic biomass [1]. Lignocellulose, the structural biopolymer found in plant cell walls, is comprised of lignin, cellulose, hemicelluloses, and to a minor extent, pectins. Due to contributions from the composition, the higher order structure of the plant cell wall, and the cellular organization of higher plants, lignocellulosic biomass is recalcitrant to many deconstruction processes that are used to release the fermentable sugars found in cellulose and hemicellulose [2, 3].

A diverse range of pretreatment technologies have been investigated that are capable of overcoming this recalcitrance, and several studies have used aqueous or organic solvents to effectively pretreat the lignocellulosic biomass for its improved conversion [4–10]. Relevant to this manuscript, significant research efforts have focused on developing pretreatments using water or ethanol as solvents in the presence of alkali [11–15]. Addition of ethanol during alkaline delignification has been found to result in more rapid delignification relative to alkaline only [16] and has been developed as a process to yield low-lignin pulps [17].

Multi-stage pretreatments offer the potential to provide a synergistic interaction in order to improve and/or generate high-yield, high-purity fractions of cell wall biopolymers and are commonly used in the forest products industry. For example, an acidic “pre-hydrolysis” coupled to an alkaline delignification is employed in the production of viscose pulps and is capable of yielding acetate and hemicellulose-derived compounds (e.g., furfural) in the first stage and a high-purity cellulose pulp suitable for the production of cellulose derivatives [18]. Comparable to these processes, autohydrolysis and dilute acid pretreatment have been coupled to a range of delignifying post-treatments in order to improve the subsequent enzymatic hydrolysis of woody biomass including hybrid poplar [19–21]. Multi-stage mild alkaline and alkaline–oxidative pretreatments have been proposed in our prior work as a method of preserving hemicellulose and maximizing enzymatic hydrolysis yields [13, 22].

We previously demonstrated that a copper-catalyzed alkaline hydrogen peroxide (Cu-AHP) pretreatment process resulted in a substantial improvement of sugar yields following enzymatic hydrolysis compared to AHP-only pretreatment [23, 24]. Further, we recently reported that the addition of an alkaline pre-extraction step prior to Cu-AHP pretreatment increased lignin and hemicellulose solubilization under mild process conditions (i.e., low temperature and pressure), improving the glucose yields by 1.4-fold (63% to 86%) and xylose yields by 1.3-fold (74% to 95%) [13]. While these data were promising, the process still utilized relatively high chemical inputs (copper, the ligand 2,2’-bipyridine (bpy) and H_2_O_2_) during pretreatment and enzyme loadings during hydrolysis to achieve the reported yields. We hypothesized that by increasing the severity of the alkaline pre-extraction step, we could increase delignification while still retaining most of the xylan, thereby allowing us to reduce both the chemical inputs required during Cu-AHP pretreatment and the enzyme loadings utilized during hydrolysis.

Although increasing the severity of alkaline pre-extraction conditions would almost certainly improve glucose yields following Cu-AHP pretreatment and enzymatic hydrolysis, an economic evaluation of pretreatment process economics is needed, as increasing the severity would also increase the processing costs. Technoeconomic analysis (TEA) evaluates both the economic and technological aspects of pretreatment technologies. In addition to understanding the total costs associated with producing ethanol from lignocellulosic feedstocks, TEA also analyzes the effects of changes in chemical input or feedstock costs, evaluates process design to maximize energy usage and recovery, and identifies process bottlenecks that might inhibit industrial-scale feasibility. As a process development tool, TEA has been applied to many pretreatment technologies including, but not limited to, dilute acid [25, 26], AFEX™ [27, 28], ionic liquid [29, 30], and γ-valerolactone (GVL) [31, 32]. Recently, TEA was applied to a two-stage alkaline hydrogen peroxide (AHP) pretreatment of corn stover, revealing a favorable minimum ethanol selling price (MESP) [33]; however, TEA on the two-stage Cu-AHP pretreatment of woody biomass has not been performed.

In this manuscript, we compare the impact that water and ethanol alkaline pre-extraction steps have on the effectiveness of Cu-AHP pretreatment of hybrid poplar. Importantly, we report that by increasing the temperature of the alkaline pre-extraction step to 120 °C, we can improve the process performance while simultaneously reducing the chemical and enzyme inputs in the second stage that are required to achieve high sugar yields following enzymatic hydrolysis. Finally, we perform economic analysis to identify areas of the pretreatment process to target for further improvements.

## Methods

### Biomass

Eighteen-year-old hybrid poplar (*Populus nigra* var. *charkoviensis *×* caudina* cv. NE-19) grown at the University of Wisconsin Arlington Agricultural Research Station was used for this study. Debarked and air-dried hybrid poplar logs were split to approximately 1.5″ × 2″ x 12″ wedges, chipped by an Earthwise 15-Amp Electric Garden Chipper/Shredder (Model GS70015), and sieved by shaking for 15 min in a LABTECH Chip Classifier with round-hole screens. Chips that passed through the 7-mm round-hole screen but were retained on the 3-mm round-hole screen were shipped to Michigan State University for use in this study.

### Compositional analysis

Prior to compositional analysis, wood chips were ground to pass through a 1-mm screen on a Christy Turner lab mill (Christy Turner LTD, Ipswich, Suffolk, UK). A two-stage acidolysis method from the National Renewable Energy Laboratory [34] was used to determine the composition of the structural carbohydrates and the acid-insoluble lignin (Klason lignin). The structural carbohydrates were separated and quantified on an Agilent 1260 series high-performance liquid chromatography (HPLC) system equipped with an Infinity II refractive index detector and an Aminex HPX-87H column. The mobile phase was 5.0 mM H_2_SO_4_ (0.6 mL/min) and the operating temperature was 65 °C. The xylose measured from the samples were reported as a cumulative percentage of xylose, mannose, and galactose as the HPX-87H column is unable to resolve these sugars.

### Alkaline pre-extraction of wood chips

For the pre-extraction step, a 5 g sample (1–2% moisture content) of 3–7 mm of hybrid poplar wood chips was heated without mixing at 120 °C for 1 h (plus a 15 min heat-up time and a 10-min cool-down time) with 250 mM NaOH (100 mg/g biomass) and either 50 mL (~ 10% wt/vol solids loadings) of 95% (vol/vol) ethanol (120 °C NaOH-EtOH pre-extraction (PE)) or water (120 °C NaOH-H_2_O PE) in a 100 mL volume capacity Parr reactor (4560 Mini Benchtop reactor). After incubation, the remaining insoluble biomass was thoroughly washed with deionized water and air dried. Prior to Cu-AHP pretreatment, the alkaline pre-extracted wood chips were milled to 1 mm using a Christy Turner lab mill (Christy Turner LTD, Ipswich, Suffolk, UK).

### Cu-AHP pretreatment

Following ethanol or water alkaline pre-extraction, the milled hybrid poplar biomass was subjected to Cu-AHP pretreatment with fed-batch addition of H_2_O_2_ [13]. The pretreatment was performed in 2.5 mL of reaction volume at 10% solids loading for 23 h at 30 °C. Unless otherwise noted, the following concentrations of reactants were utilized during pretreatment. Catalyst loadings were set at 1 mM for Cu^2+^ (added as CuSO_4_·5H_2_O) and 2 mM for 2,2′-bipyridine (bpy), respectively. (Although the precise function of the bpy ligand is unknown, the N heteroatom donor and the aromaticity of the bidentate ligand are important.) The H_2_O_2_ and NaOH loadings were both set at 100 mg/g of hybrid poplar biomass. Fed-batch addition of H_2_O_2_ was performed over a 10-h period to achieve a final oxidant loading of 100 mg/g pre-extracted biomass.

### Enzymatic hydrolysis

Following 23 h of Cu-AHP pretreatment, the pH of the pretreatment mixture was adjusted to 5.0 with 72% (w/w) H_2_SO_4_ followed by the addition of 0.25 mL of 1 M citric acid buffer (pH 5.0). To complete enzymatic hydrolysis, Cellic CTec3 (197.3 mg/g) and HTec3 (170.5 mg/g), provided by Novozymes A/S (Bagsværd, DK), were added into the reaction mixture, each at a loading of 15 mg protein/g glucan from pre-extracted biomass, for a total protein loading of 30 mg/g. The enzyme content was supplied by the manufacturer. The total aqueous volume of the reaction was then adjusted to 5 mL by adding deionized water to attain solids loading of 5% (wt/vol). The samples were incubated at 50 °C for 72 h with orbital shaking at 210 rpm. The sugars obtained following enzymatic hydrolysis were quantified by high-performance liquid chromatography following a procedure described previously [13]. The sugar yields (glucose and xylose) were calculated by dividing the amount of released sugar by the total sugar content of the biomass (dry weight basis) prior to pretreatment.

### Technoeconomic analysis

A technoeconomic analysis (TEA) based on n^th^ plant assumptions was performed to assess economic improvements resulting from the two-stage Cu-AHP pretreatment conditions that were evaluated experimentally. Three processes were considered to precede Cu-AHP pretreatment: 1) 30 °C NaOH-H_2_O PE [13], 2) 120 °C NaOH-H_2_O PE (as described above), and 3) 120 °C NaOH-EtOH PE (as described above). Process flow diagrams of each process and biorefinery are provided in Additional file 1: Figure S1 and S2. After pre-extraction, solids are separated from liquids by filtering through screens at the bottom of the pre-extraction reactor. These solids are sent to the Cu-AHP pretreatment reactors, while the liquor containing base and solubilized lignin, xylan, glucan, acetate, and mineral ash is sent to a Kraft paper mill, serving as an integrated biorefinery, to partially recover NaOH for use in the Kraft mill. NaOH needed by the biorefinery is assumed to be purchased at its market price whether from the adjacent Kraft mill or from a separate vendor. For 120 °C NaOH-EtOH PE, the ethanol-rich liquor is sent to a distillation column to recover ethanol and recycle it to the pre-extraction reactors. The bottoms of ethanol recovery column are routed to the integrated Kraft paper mill as a means of handling the waste that contains NaOH. Further information on operating conditions and conversions are provided as supplementary data (see Additional file 1: TableS2, Additional file 1: Figures S1, S2). Aspen Plus Version 8.8 was used to simulate material and energy balances for the three process configurations.

The 2011 NREL biorefinery model [35], designed for bioethanol production from corn stover, served as the basis scenario for each simulation. The model was updated to utilize poplar at 20% moisture content and produce 60 million (MM) gal/year of ethanol. The bench-scale experiments performed in this study provided the processing conditions and yields that were implemented by the biorefinery model. Capital costs for each area of the biorefinery model were primarily scaled from the NREL equipment cost estimates in the 2011 [35], 2013 [36], and 2015 reports [37]. These were then adjusted to 2011 dollars using the Chemical Engineering Plant Cost Index, the primary source of which is Chemical Engineering Magazine, to allow for comparison with recent NREL reports [35–37]. The operating hours of the biorefinery were changed to 7880 h per year. Raw material prices are also updated to 2011 dollars using the Industrial Inorganic Chemical Index (see Additional file 1 Table S1) [38]. A unit production cost (UPC) was calculated for each process based on the capital and operating costs (Eqs. 1–3) [39]. The calculated UPC represents the minimum selling price of ethanol to compensate for the annual production cost of the biorefinery,

1 $$ {\text{UPC}} = \left( {{\text{ACC}} + {\text{TOC}}} \right)/{\text{APR}}, $$

2 $$ {\text{ACC}}\,{ = }\,{\text{CCF}} \times C_{\text{P}}, $$

3 $$ {\text{CCF}} = [r ( 1 + r )^{\text{n}} / [ ( 1 + r )^{\text{n}} - 1], $$

where ACC, TOC, and APR are annualized capital cost, total operating costs, and annual ethanol production rate (60 MMgal/year), respectively. *C*_P_ is the total capital cost and CCF is the capital charge factor calculated to be 0.1061 for an interest rate (*r*) of 10% and a plant life of 30 years. OC is calculated as the summation of raw material and fixed operating costs minus revenues from selling byproducts. Annual fixed operating costs are assumed to be 5.1% of total capital costs [39].

## Results and discussion

We have previously demonstrated the improved hydrolysis yields of the Cu-AHP pretreatment process in treating hybrid poplar compared to both alkaline-only and alkaline hydrogen peroxide-only treatments when all pretreatments were conducted at 30 °C [12, 23, 24]. Additionally, we demonstrated that adding an alkaline pre-extraction step at 30 °C to the Cu-AHP pretreatment resulted in an increase in glucose and xylose yields by 23 and 21%, respectively [13].

Based on this promising yield increase, we hypothesized that by increasing severity of the alkaline pre-extraction, we could further reduce the chemical costs during Cu-AHP while still maintaining high sugar yields. Therefore, the current study focused on further improvements to Cu-AHP pretreatment by performing alkaline pre-extraction at high temperature in two different solvent systems, *i.e.*, ethanol and water. Additionally, we performed the alkaline pre-extraction step on larger sized poplar wood chips (3–7 mm) that were then milled to 20 mesh (0.85 mm) screen size using a Wiley mill prior to Cu-AHP pretreatment. Studies have shown that pretreating wood chips prior to milling to a size appropriate for enzymatic hydrolysis can reduce energy consumption compared to milling prior to pretreatment [40, 41].

**Fig. 1:**
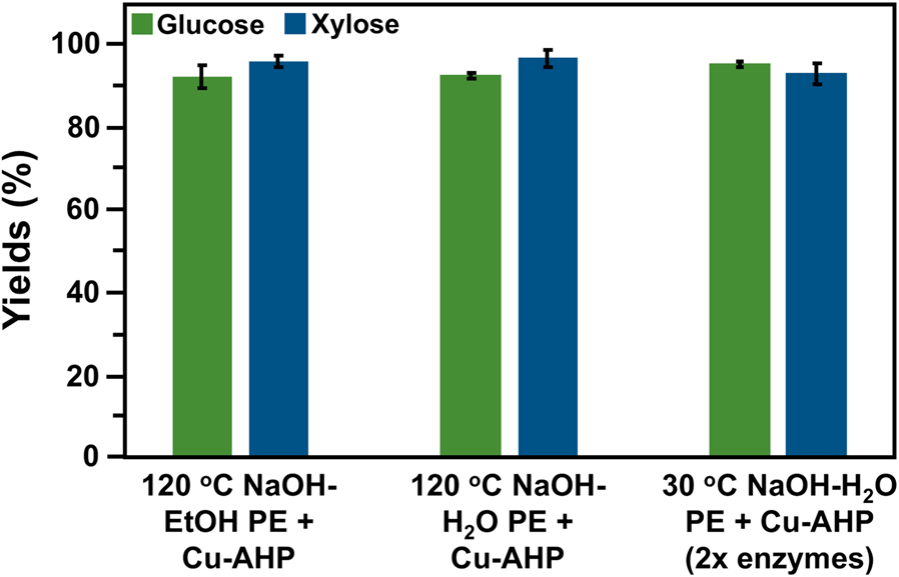
Glucose and xylose yields following enzymatic hydrolysis of alkaline pre-extracted (in water or ethanol) and fed-batch Cu-AHP pretreated hybrid poplar (yields based on composition of alkaline pre-extracted biomass). All pretreatment reactions were performed for 24 h at 10% (w/v) solids. For the two 120 °C pre-extractions completed as part of this study, particle size was 3–7 mm for pre-extraction and 1 mm for Cu-AHP pretreatment. The temperature for pretreatment was 30 °C, with final concentrations of 1 mM Cu^2+^ and 2 mM bpy, and a H_2_O loading of 100 mg/g biomass. Enzyme loadings for enzymatic hydrolysis were 30 mg total protein per g glucan. The 30 °C pre-extraction was from a previous study [13]. Experimental conditions were largely the same as above, except that samples were milled to pass through a 20-mesh screen (0.85 mm) prior to pre-extraction, and enzyme loadings of 60 mg total protein per g glucan. The data points are the averages of three independent experiments, and the error bars represent ± standard deviations of the means

To test the hypothesis that increasing the temperature of the alkaline pre-extraction would allow us to reduce inputs during Cu-AHP, hybrid poplar biomass was mixed with the solvent (ethanol or water) in the presence of alkali and incubated at 120 °C for 1 h instead of at 30 °C, as in our previous studies. After completing Cu-AHP pretreatment and enzymatic hydrolysis, we observed between 93 and 98% conversion of glucan and xylan of pre-extracted biomass to glucose and xylose (Fig. 1).

Compositional analysis of the pretreated biomass was performed to determine the changes associated with the pre-extraction treatments relative to untreated biomass. A larger amount of mass was solubilized during 120 °C NaOH-H_2_O PE (~ 21%) compared to 120 °C NaOH-EtOH PE (~ 16%). Compositional analysis of treated biomass demonstrated that ~ 27% of the original xylan and ~ 28% of the original lignin was solubilized during 120 °C NaOH-H_2_O PE compared to ~ 20% xylan and 19% lignin solubilization for 120 °C NaOH-EtOH PE-treated biomass (see Additional file 1: Table S2). In comparison, only ~ 5% of both the lignin and xylan were solubilized during 30 °C NaOH-H_2_O PE [13].

While both 120 °C pre-extraction steps resulted in some lignin removal, the majority of the delignification occurred when the pre-extracted biomass was further subjected to Cu-AHP pretreatment. After Cu-AHP pretreatment of 120 °C NaOH-H_2_O PE and 120 °C NaOH-EtOH PE biomass, 78% and 74%, respectively, of the original lignin content was removed from a combination of pre-extraction and pretreatment. This is an approximate 1.4-fold increase in lignin solubilization compared to the 30 °C NaOH-H_2_O PE (55%) [13].

**Fig. 2:**
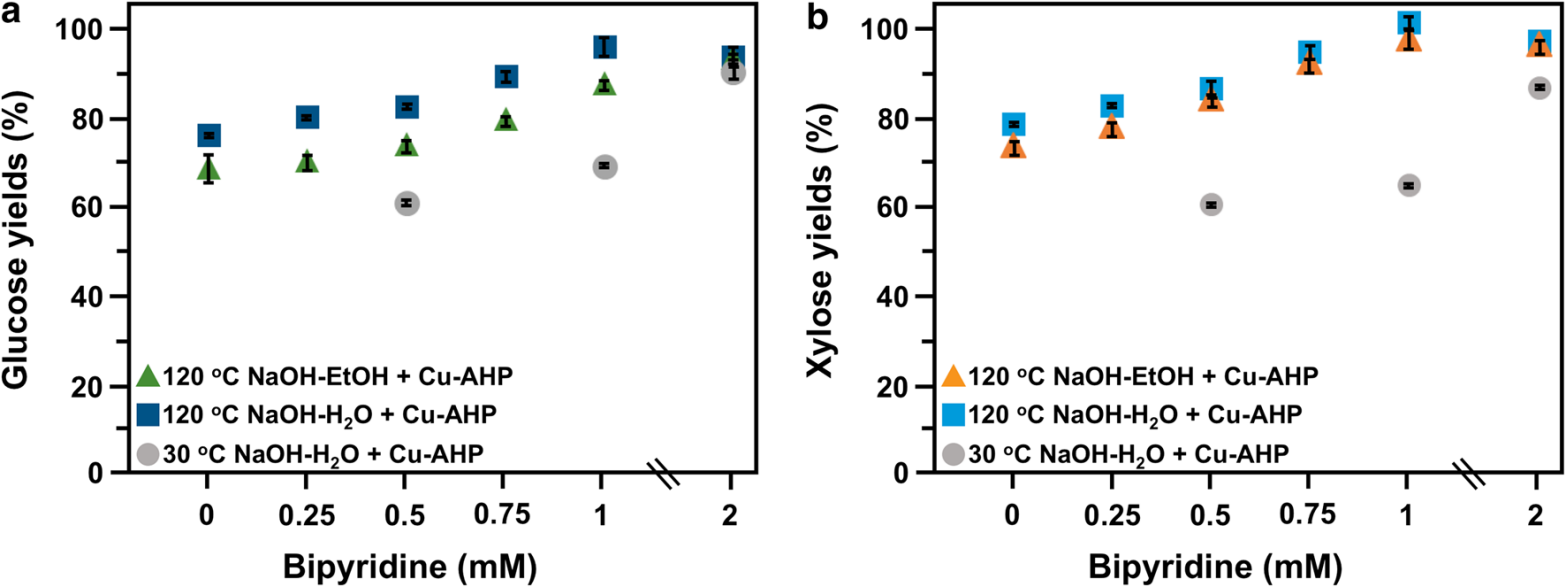
Glucose (**a**) and xylose (**b**) yields following enzymatic hydrolysis of alkaline pre-extracted and fed-batch Cu-AHP pretreated hybrid poplar at different bpy loadings (yields based on composition of alkaline pre-extracted biomass). Triangles represent 120 °C NaOH-EtOH pre-extraction and squares represent 120 °C NaOH-H_2_O pre-extraction. Pretreatment reactions were performed at 30 °C for 24 h at 10% (w/v) solids. Particle size was 3–7 mm for pre-extraction and 1 mm for Cu-AHP pretreatment. The final concentrations in the reaction were 1 mM Cu^2+^ and 100 mg/g biomass for the H_2_O_2_. Enzyme loadings for enzymatic hydrolysis were 30 mg total protein per g glucan. The data points are the averages of three independent experiments, and the error bars represent ± standard deviations of the means. Circles represent 30 °C NaOH-H_2_O pre-extraction from a previous study [13]. Experimental conditions were largely the same as above, except that samples were milled to pass through a 20-mesh screen (0.85 mm) prior to pre-extraction, and a temperature of 30 °C during both pre-extraction and pretreatment. The data points are the averages of three independent experiments, and the error bars represent ± standard deviations of the means

We then performed a series of experiments to determine if the increase in lignin solubilization caused by the high-temperature alkaline pre-extraction would allow chemical and enzyme loadings to be reduced without negatively impacting final sugar yields. A preliminary cost analysis indicated that bpy, H_2_O_2_, enzymes, and NaOH are the major raw material costs (other than feedstocks) in the overall conversion process. Therefore, the first set of experiments were carried out at reduced bpy loadings while Cu^2+^ concentrations were maintained at 1 mM. High glucose yields (~ 90%) were still observed when the bpy concentration was reduced to 0.75 mM with 120 °C NaOH-H_2_O PE/Cu-AHP, but slightly lower yields (~ 80%) were noted for 120 °C NaOH-EtOH PE/Cu-AHP (Fig. 2). Further, when pre-extraction was carried out at 120 °C, the glucose yields obtained when ethanol was used with 0.75 mM bpy were the same as when water was used with just 0.5 mM bpy. Interestingly, when no bpy was added during the Cu-AHP treatment, glucose yields were 76% when water was used during pre-extraction and~ 70% when ethanol was used. Overall, the results showed significant improvements in sugar yields over 30 °C NaOH-H_2_O PE/Cu-AHP [13] where glucose yields dropped to~ 70% when bpy was reduced to 0.5 mM concentration.

**Fig. 3:**
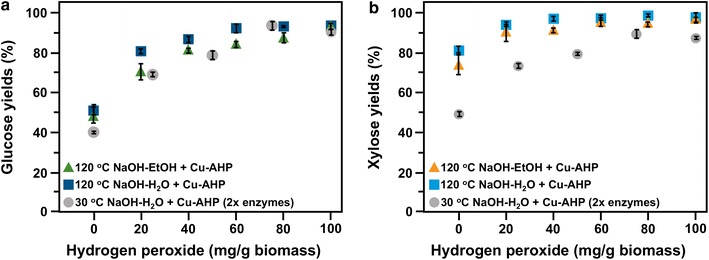
Glucose (**a**) and xylose (**b**) yields following enzymatic hydrolysis of alkaline pre-extracted and fed-batch Cu-AHP pretreated hybrid poplar at different H_2_O_2_ loadings (yields based on composition of alkaline pre-extracted biomass). Triangles represent 120 °C NaOH-EtOH pre-extraction and squares represent 120 °C NaOH-H_2_O pre-extraction. Pretreatment reactions were performed at 30 °C for 24 h at 10% (w/v) solids. Particle size was 3–7 mm for pre-extraction and 1 mm for Cu-AHP pretreatment. The final Cu^2+^ and bpy concentrations in the reaction were 1 mM and 2 mM, respectively. Enzyme loadings for enzymatic hydrolysis were 30 mg total protein per g glucan. The data points are the averages of three independent experiments, and the error bars represent ± standard deviations of the means. Circles represent 30 °C NaOH-H_2_O pre-extraction from a previous study [13]. Experimental conditions were largely the same as above, except that samples were milled to pass through a 20-mesh screen (0.85 mm) prior to pre-extraction, and a temperature of 30 °C during both pre-extraction and pretreatment, and enzyme loadings of 60 mg total protein per g glucan. The data points are the averages of three independent experiments, and the error bars represent ± standard deviations of the means

In the second set of experiments, we reduced H_2_O_2_ loadings while bpy (2 mM), Cu^2+^ (1 mM) and enzymes loadings (30 mg/g original glucan) were kept constant (Fig. 3). The results demonstrated that 120 °C NaOH-H_2_O PE/Cu-AHP resulted in slightly higher glucose yields (3–10% increase) and xylose yields (2–6% increase) compared to 120 °C NaOH-EtOH PE/Cu-AHP at all peroxide loadings. Further, H_2_O_2_ could be reduced to 40 mg/g biomass while still maintaining high glucose yields for both 120 °C NaOH-H_2_O PE/Cu-AHP (86%) and 120 °C NaOH-EtOH PE/Cu-AHP (81%). The biomass treated with just 20 mg H_2_O_2_/g biomass and subjected to 120 °C NaOH-H_2_O PE/Cu-AHP still resulted in just over 80% glucose yields. The complete elimination of H_2_O_2_, however, resulted in only ~ 50% glucose yields for both 120 °C NaOH-H_2_O PE and 120 °C NaOH-EtOH PE.

**Fig. 4:**
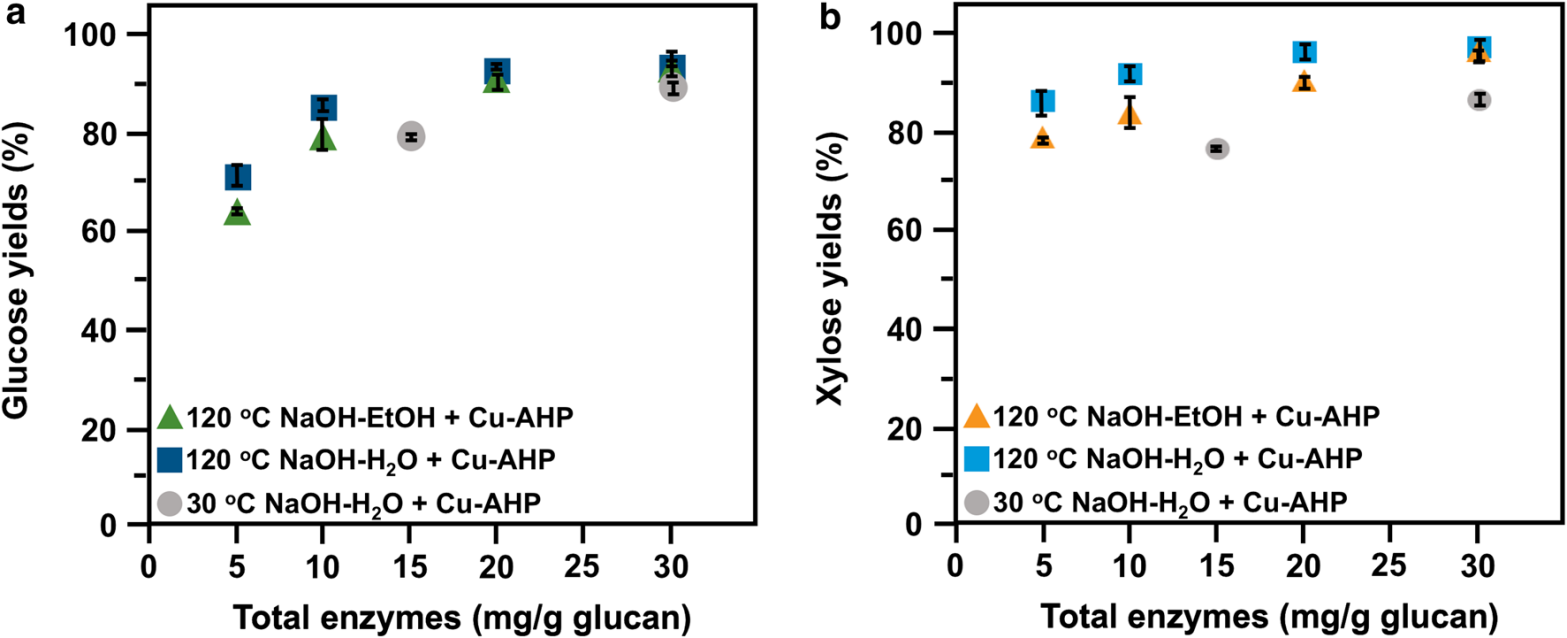
Glucose (**a**) and xylose (**b**) yields following enzymatic hydrolysis of alkaline pre-extracted and fed-batch Cu-AHP pretreated hybrid poplar at different total enzyme loadings (yields based on composition of alkaline pre-extracted biomass). Triangles represent 120 °C NaOH-EtOH pre-extraction and squares represent 120 °C NaOH-H_2_O pre-extraction. Pretreatment reactions were performed at 30 °C for 24 h at 10% (w/v) solids. Particle size was 3–7 mm for pre-extraction and 1 mm for Cu-AHP pretreatment. The final Cu^2+^ and bpy concentrations in the reaction were 1 mM and 2 mM, respectively. The final H_2_O_2_ concentration was 100 mg/g biomass. The data points are the averages of three independent experiments, and the error bars represent ± standard deviations of the means. Circles represent 30 °C NaOH-H_2_O pre-extraction from a previous study [13]. Experimental conditions were largely the same as above, except that samples were milled to pass through a 20-mesh screen (0.85 mm) prior to pre-extraction and a temperature of 30 °C during both pre-extraction and pretreatment. The data points are the averages of three independent experiments, and the error bars represent ± standard deviations of the means

We performed a third set of experiments where total enzyme loadings were reduced, although the ratio of Cellic CTec3:HTec3 remained 1:1, while H_2_O_2_ (100 mg/g glucan), Cu^2+^ (1 mM) and bpy loadings (2 mM) were held constant (Fig. 4). At a total enzyme loading of 20 mg/g glucan (10 mg/g each protein), glucose yields of > 90% were still achieved for both 120 °C NaOH-H_2_O PE and 120 °C NaOH-EtOH PE. The results also revealed that at lower total enzyme loadings of 10 and 5 mg/g glucan, the glucose yields were higher for 120 °C NaOH-H_2_O PE/Cu-AHP compared to the 120 °C NaOH-EtOH PE/Cu-AHP.

### Technoeconomic analysis results

Increasing the temperature of the alkaline PE step to 120 °C allowed for a decrease in bpy, H_2_O_2_, and enzymes with a small variation in glucose and xylose yields compared to 30 °C alkaline PE. However, 120 °C alkaline PE requires heat input at an economic cost. Therefore, this preliminary TEA was performed to gauge how process changes improve the biorefinery economics.

Simulation results were used to calculate the raw material and variable operating cost of each process. A detailed list of the annual raw material cost for the three processes, *i.e.*, 30 °C NaOH-H_2_O water PE/Cu-AHP [13], 120 °C NaOH-H_2_O PE/Cu-AHP, and 120 °C NaOH-EtOH PE/Cu-AHP, is provided in Table S3 (see Additional file 1: Table S3). Total raw material costs for 30 °C NaOH-H_2_O PE, 120 °C NaOH-H_2_O PE, and 120 °C NaOH-EtOH PE were calculated to be $197.6, $175.3, and $191.8 MM/year, respectively. Other than feedstocks, H_2_O_2_, bpy, enzymes, and NaOH comprise the main raw material costs in the pretreatment unit in all cases. Smaller carbohydrate losses in 30 °C NaOH-H_2_O PE translate to less biomass required for making 60 MMgal/year ethanol, which reduces biomass cost by about $3 MM/year. Further, 30 °C NaOH-H_2_O PE and 120 °C NaOH-H_2_O PE produce electricity as a by-product by burning lignin and have an annual revenue of $2.82 and $3.63 MM/year from selling electricity to the grid. Additionally, 30 °C NaOH-H_2_O PE consumes more power for aeration and agitation in cellulase enzyme production vessels as the enzyme requirement of 30 °C NaOH-H_2_O PE is twice that of 120 °C NaOH-H_2_O PE and 120 °C NaOH-EtOH PE (60 vs 30 mg/g glucan). Increased enzymes also lead to increased sugar costs for onsite enzyme production, as 30 °C NaOH-H_2_O PE sugar cost ($48.2 MM/year) is about double that of 120 °C NaOH-H_2_O PE ($27.1 MM/year) and 120 °C NaOH-EtOH PE ($26.1 MM/year). Finally, 120 °C NaOH-EtOH PE requires a larger amount of heat to recover ethanol used in the pre-extraction reactors. More troubling, the heat obtained from combusting the solid residues of fermentation and the biogas from wastewater treatment is insufficient to meet the heat demands, requiring that purchased natural gas be burned (about $1.84 MM/year) to provide the necessary process heat. Consequently, 120 °C NaOH-EtOH PE does not produce excess steam for power production and must purchase grid electricity costing $12.3 MM/year.

**Fig. 5:**
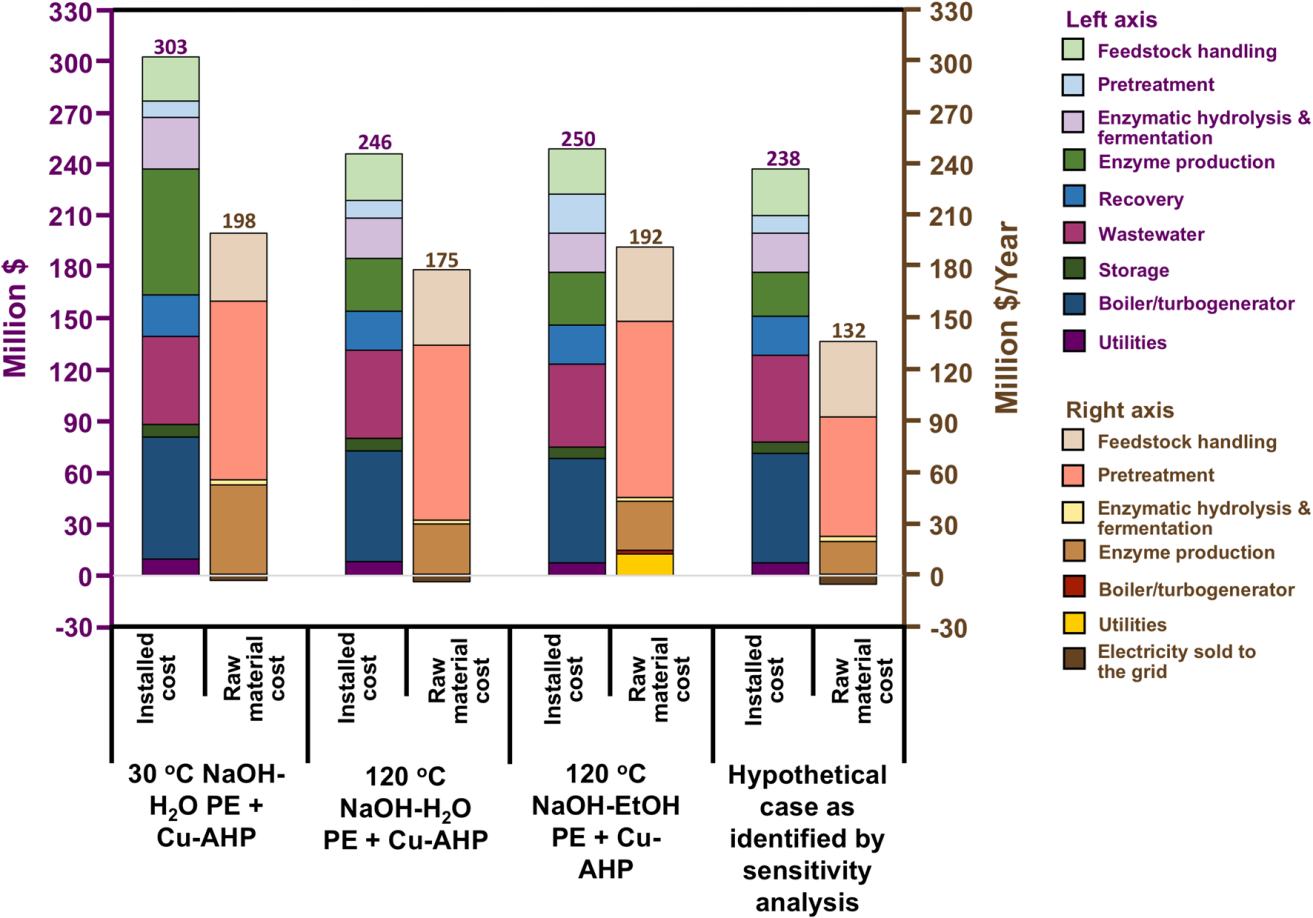
Comparison of installed costs and raw material costs between the three process scenarios considered by TEA. These scenarios are then compared to a “hypothetical case” 120 °C NaOH-H_2_O pre-extraction process that reduces H_2_O_2_, enzyme, and bpy loadings in concert. Note that raw material costs in this figure include the by-product revenue from electricity sales

A detailed itemization of the total capital investment (TCI) for each process is shown in Table S4 (see Additional file 1 Table S4). The installed cost of the pretreatment unit in 120 °C NaOH-EtOH PE is about $13 MM more expensive than 30 °C NaOH-H_2_O PE and 120 °C NaOH-H_2_O PE because of the capital costs attributed to ethanol recovery, the reboiler, and air-cooled condensers. Overall, 30 °C NaOH-H_2_O PE resulted in $100 MM higher capital costs than the other processes, primarily because of higher capital costs for enzyme production. Consequently, UPC for 30 °C NaOH-H_2_O PE/Cu-AHP, 120 °C NaOH-H_2_O PE/Cu-AHP, and 120 °C NaOH-EtOH PE/Cu-AHP was calculated as 4.09, 3.57, and 3.85 $/gal, respectively. This indicates that compared to the other process, 120 °C NaOH-H_2_O PE has the better economics because of its lower operating and investment cost (Fig. 5).

Using the 120 °C NaOH-H_2_O PE model, a sensitivity analysis, based on the actual bench-scale data obtained by this work, was performed to assess the potential for further cost reduction. Experimental data show that H_2_O_2_, enzyme, and bpy loadings can be reduced from their base value of 100 mg/g dry biomass (to 60 mg/g dry biomass), 30 mg/g glucan (to 20 mg/g glucan), and 2 mM (to 1 mM), respectively, without meaningfully lowering the glucose and xylose yields. To gauge the impact of altering these three variables on process economics, a hypothetical case was formulated by assuming no interaction effects. If H_2_O_2_, enzyme, and bpy loadings can be reduced in concert without lowering the fermentable sugar yield, then the overall impact of these changes reduced the total raw material cost of the process and capital costs of the enzyme production unit by about 25% and 16% to $131.7 MM/year and $25.6 MM, respectively. The excess electricity sold to the grid also increased from $3.6 to $5.2 MM/year, a 44% increase. Overall, these changes reduced UPC from $3.57 to $2.82/gal, which is a 21% decrease. Figure 5 compares the three cases examined in this study to a 120 °C NaOH-H_2_O PE hypothetical case that benefits from lowering H_2_O_2_, enzyme, and bpy loadings in concert. As expected, reductions in raw materials used by 120 °C NaOH-H_2_O PE results in significant reductions in operating costs, and to a lesser degree, capital costs. Of course, interaction effects could alter these predictions positively or negatively; future experiments should be designed to simultaneously vary multiple factors and measure the influence of interaction on costs.

Another possible option to further reduce the raw material cost would be lowering the current bpy price ($59/kg) by mass production. A five- and ten-fold reduction in bpy cost will reduce the annual raw material cost for high-temperature water PE by about $22.7 and $25.5 MM/year, respectively, which is a significant decrease. However, this option assumes future economies of scale for the production of bpy. Therefore, scenarios that include bpy recycling or onsite production should be considered in future studies.

A complete life cycle analysis, which would provide a more detailed picture of the environmental impact of the three process configurations, was not performed. However, several observations can be made based solely on the mass and energy balances. For example, as a consequence of the higher electricity and natural gas inputs, the process utilizing 120 °C NaOH-EtOH PE would result in higher fossil fuel input and greenhouse gas emissions per unit of biofuel generated than either of the other two process configurations. On the other hand, the 120 °C NaOH-EtOH PE process requires less process water per unit of biofuel produced. Further analysis is needed to fully understand these tradeoffs.

## Conclusion

In conclusion, we found that increasing the temperature of the alkaline pre-extraction step to 120 °C prior to Cu-AHP pretreatment allowed bpy, H_2_O_2_, and enzyme loadings to be decreased without causing as large of a reduction in glucose and xylose yields as was seen with a 30 °C NaOH-H_2_O PE. Additionally, sugar yields with 120 °C NaOH-H_2_O PE were greater than those for 120 °C NaOH-EtOH PE. TEA revealed that 30 °C NaOH-H_2_O PE was the most expensive of the three pre-extraction methods, and that 120 °C NaOH-H_2_O PE had the lowest investment and operating costs. Further experimental work, coupled with TEA, will be required to identify the optimal severity of the alkaline pre-extraction step relative to the Cu-AHP extraction and the reduction of non-feedstock inputs.

## Additional file

**Additional file 1: Table S1.** Raw material prices considered in the technoeconomic analysis**. Table S2.** Operating conditions considered for technoeconomic assessment of industrial scale bioethanol plant utilizing two stage Cu-AHP preteatments. The first three columns use data that were collected during this investigation. Entries in the fourth column, for the best-case, assume that glucose and xylose yields from the “120 °C NaOH-H_2_O PE + Cu-AHP case” remain unchanged when hydrogen peroxide, 2,2ʹ-bipyridine (bpy), and enzyme loadings are reduced. **Table S3.** Comparison of raw material costs (MM $/year) for 60 MM gal/year bioethanol plant utilizing two-stage Cu-AHP pretreatments. **Table S4.** Total capital investments of 60 MM gal/year bioethanol plant utilizing two-stage Cu-AHP pretreatments. **Figure S1.** Block flow diagram of the biorefinery. **Figure S2.** Process flow diagram of pretreatment unit including first stage (a) 30 °C NaOH-H_2_O PE, (b) 120 °C NaOH-H_2_O PE, and (c) 120 °C NaOH-EtOH PE followed by second stage Cu-AHP pretreatment.

## Abbreviations

30 °C NaOH-H_2_O PE: alkaline pre-extraction step conducted in water at 30 °C
120 °C NaOH-EtOH PE: alkaline pre-extraction step conducted in ethanol at 120 °C
120 °C NaOH-H_2_O PE: alkaline pre-extraction step conducted in water at 120 °C
ACC: annualized capital cost
APR: annual ethanol production rate
bpy: 2,2′-bipyridine
CCF: capital charge factor
*C*_P_: total capital cost
Cu-AHP: copper-catalyzed alkaline hydrogen peroxide
GVL: γ-valerolactone
HPLC: high-performance liquid chromatography
PE: pre-extraction
TEA: technoeconomic analysis
TOC: total operating costs
UPC: unit production cost
