# Supplementary material for: Integrated experimental and technoeconomic evaluation of two-stage Cu-catalyzed alkaline–oxidative pretreatment of hybrid poplar
Source: Biotechnol Biofuels. 2018 May 17;11:143. doi: 10.1186/s13068-018-1124-x (PMC5956811; doi:10.1186/s13068-018-1124-x)
Supplement: Supplementary file 1 — Additional file 1: Table S1. Raw material prices considered in the technoeconomic analysis. Table S2. Operating conditions considered for technoeconomic assessment of industrial scale bioethanol plant utilizing two stage Cu-AHP preteatments. The first three columns use data that were collected during this investigation. Entries in the fourth column, for the best-case, assume that glucose and xylose yields from the “120 °C NaOH-H2O PE + Cu-AHP case” remain unchanged when hydrogen peroxide, 2,2ʹ-bipyridine (bpy), and enzyme loadings are reduced. Table S3. Comparison of raw material costs (MM $/year) for 60 MM gal/year bioethanol plant utilizing two-stage Cu-AHP pretreatments. Table S4. Total capital investments of 60 MM gal/year bioethanol plant utilizing two-stage Cu-AHP pretreatments. Figure S1. Block flow diagram of the biorefinery. Figure S2. Process flow diagram of pretreatment unit including first stage (a) 30 °C NaOH-H2O PE, (b) 120 °C NaOH-H2O PE, and (c) 120 °C NaOH-EtOH PE followed by second stage Cu-AHP pretreatment. [file 13068_2018_1124_MOESM1_ESM.docx]

Additional Files

**Integrated experimental and technoeconomic evaluation of two-stage Cu-catalyzed alkaline-oxidative pretreatment of hybrid poplar**

Aditya Bhalla^1,2,†^, Peyman Fasahati^1,3^, Chrislyn A. Particka^1^, Aline E. Assad^1^, Ryan J. Stoklosa^1,4^, Namita Bansal^1,2^, Rachel Semaan^2^, Christopher M. Saffron^1,3,4,^*, David B. Hodge^1,3,4,5,^*, Eric L. Hegg^1,2,^*

^1^ DOE Great Lakes Bioenergy Research Center, Michigan State University, 1129 Farm Lane, East Lansing, MI 48824, USA

^2^ Department of Biochemistry & Molecular Biology, Michigan State University, 603 Wilson Road, East Lansing, MI 48824, USA

^3^ Department of Biosystems & Agricultural Engineering, Michigan State University, 216 Farrall Hall, East Lansing, MI 48824, USA

^4^ Department of Chemical Engineering & Materials Science, Michigan State University, 428 S. Shaw Lane, East Lansing, MI 48824, USA

^5^ Division of Sustainable Process Engineering, Luleå University of Technology, SE-98187, Luleå, Sweden

A. Bhalla – bhalla@msu.edu

P. Fasahati – fasahati@wisc.edu

C. Particka – chrislyn@msu.edu

A. Assad - alineeasilva@yahoo.com.br

R. Stoklosa – ryan.stoklosa@ars.usda.gov

N. Bansal – namita@msu.edu

R. Semann – semaanra@msu.edu

*C. Saffron – saffronc@msu.edu, corresponding author

*D. Hodge – david.hodge3@montana.edu, corresponding author

*E. Hegg – erichegg@msu.edu, corresponding author

Table S1. Raw material prices considered in the technoeconomic analysis.

| **Raw Material** | **2011 Price  $/U.S. ton** | **References** |
| --- | --- | --- |
| Sulfuric Acid, 93% | 99.8 | [1] |
| Corn Steep Liquor | 63 | [2] |
| Diammonium Phosphate | 1,097 | [2] |
| Sorbitol | 1,253 | [2] |
| Glucose | 645 | [2] |
| Host nutrients | 913 | [2] |
| Sulfur Dioxide | 338 | [2] |
| Boiler Chemicals | 5,557 | [2] |
| Lime | 222 | [2] |
| Natural Gas | 227 | [2] |
| Cooling Tower Chemicals | 3,330 | [2] |
| Electricity ($/kWh) | 0.0572 | [2] |
| Makeup Water | 0.29 | [2] |
| Caustic (As pure) | 166 | [3] |
| Feedstock (poplar, dry basis) | 60 | [4] |
| Hydrogen Peroxide (50%) | 363 | [5] |
| Copper Sulfate | 27 | [6] |
| BPY (2,2'-Bipyridine) | 53,520 | [7] |

Table S2. Operating conditions considered for technoeconomic assessment of industrial scale bioethanol plant utilizing two stage Cu-AHP preteatments. The first three columns use data that were collected during this investigation. Entries in the fourth column, for the best-case, assume that glucose and xylose yields from the “120 °C NaOH-H_2_O PE + Cu-AHP case” remain unchanged when hydrogen peroxide, 2,2ʹ-bipyridine (bpy), and enzyme loadings are reduced.

|  | 30 °C NaOH-H_2_O PE + Cu-AHP | | | 120 °C NaOH-H_2_O PE + Cu-AHP | | 120 °C NaOH-EtOH PE + Cu-AHP | | Hypothetical-case as identified by sensitivity analysis | |
| --- | --- | --- | --- | --- | --- | --- | --- | --- | --- |
| **1^st^ stage pre-extraction** |  | | | | | | | |  |
| Temperature (ᵒC) | 30 °C | | | 120 °C | | 120 °C | | 120 °C | |
| NaOH (g/g dry biomass) | 0.1 | | | 0.1 | | 0.1 | | 0.1 | |
| Solids loading^a^ | 30% | | | 30% | | 30% | | 30% | |
| Dissolved xylan^b^ | 5% | | | 27% | | 20% | | 27% | |
| Dissolved cellulose^b^ | 1% | | | 4% | | 2 | | 4% | |
| Dissolved lignin^b^ | 5% | | | 28% | | 19% | | 28% | |
| Residence time | 1 (hr) | | | 1 (hr) | | 1 (hr) | | 1 (hr) | |
| **2^nd^ stage pretreatment** |  | | | | | | | |  |
| Temperature | 30 °C | | | 30 °C | | 30 °C | | 30 °C | |
| NaOH (g/g dry biomass) | 0.1 | | | 0.1 | | 0.1 | | 0.1 | |
| H_2_O_2_ (g/g dry biomass) | 0.1 | | | 0.1 | | 0.1 | | 0.06 | |
| CuSO_4_ (mM) | 1 | | | 1 | | 1 | | 1 | |
| bpy (mM) | 2 | | | 2 | | 2 | | 1 | |
| Dissolved xylan^c^ | 37% | | | 25% | | 25% | | 25% | |
| Dissolved cellulose ^c^ | 5% | | | 1% | | 3% | | 1% | |
| Dissolved lignin ^c^ | 53% | | | 70% | | 68% | | 70% | |
| Residence time | 23 (hr) | | | 23 (hr) | | 23 (hr) | | 23 (hr) | |
| Solids loading^*^ | 30% | | | 30% | | 30% | | 30% | |
| **Net lignin removal** ^d^ | **55%** | | | **78%** | | **74%** | | **78%** | |
| **Enzymatic hydrolysis** |  | |  | |  | |  | |  |
| Glucose yield | 96% | | | 93% | | 93% | | 93% | |
| Xylose yield | 94% | | | 98% | | 97% | | 98% | |
| Time (hr) | 72 | | | 72 | | 72 | | 72 | |
| Enzyme loading (mg protein/g glucan) | 60 | | | 30 | | 30 | | 20 | |
| Solids loading^a^ | 20% | | | 20% | | 20% | | 20% | |
| Temperature (ᵒC) | 50 | | | 50 | | 50 | | 50 | |
| **Fermentation** |  | | |  | |  | |  | |
| Temperature (ᵒC)^a^ | 32 | | | 32 | | 32 | | 32 | |
| Time (hr)^a^ | 36 | | | 36 | | 36 | | 36 | |
| Glucose to EtOH conversion | | 95% | | 95% | | 95% | | 95% | |
| Xylose to EtOH conversion | 85% | | | 85% | | 85% | | 85% | |

^a^ Assumed based on NREL report ‎[1].

^b^ Grams dissolved constituent/grams constituent in original biomass.

^c^ Grams dissolved constituent/grams constituent in pre-extracted biomass.

^d^ The net lignin dissolved cannot be directly computed by addition of the 1^st^ stage pre-extraction and 2^nd^ stage pretreatment because the basis units differ.

Table S3. Comparison of raw material costs (MM $/year) for 60 MM gal/year bioethanol plant utilizing two-stage Cu-AHP pretreatments.

| Process Area | 30 °C NaOH-H_2_O PE + Cu-AHP  MM$/year | 120 °C NaOH-H_2_O PE + Cu-AHP  MM$/year | 120 °C NaOH-EtOH PE + Cu-AHP  MM$/year | Hypothetical-case  as identified by sensitivity analysis  MM$/year |
| --- | --- | --- | --- | --- |
| Area 100: Feedstock handling |  |  |  |  |
| Feedstock | 40.22 | 44.46 | 43.17 | 44.45 |
| Area 200: Pretreatment |  |  |  |  |
| Sulfuric acid, 93% | 8.17 | 7.85 | 8.01 | 7.84 |
| Sodium hydroxide | 21.49 | 22.31 | 22.07 | 22.31 |
| Hydrogen peroxide | 45.08 | 43.27 | 44.16 | 25.95 |
| Copper sulfate | 0.01 | 0.01 | 0.01 | 0.01 |
| Bipyridine | 29.51 | 28.34 | 28.95 | 13.70 |
| Area 300: Enzymatic Hydrolysis & Fermentation |  |  |  |  |
| Corn steep liquor | 0.63 | 0.63 | 0.58 | 0.60 |
| Diammonium phosphate | 1.37 | 1.36 | 1.26 | 1.31 |
| Sorbitol | 0.47 | 0.47 | 0.43 | 0.45 |
| Area 400: Enzyme Production |  |  |  |  |
| Glucose | 48.23 | 27.07 | 26.11 | 18.04 |
| Corn steep liquor | 0.32 | 0.18 | 0.17 | 0.12 |
| Ammonia | 1.77 | 1.00 | 0.96 | 0.66 |
| Host nutrients | 1.90 | 1.07 | 1.03 | 0.71 |
| Sulfur dioxide | 0.17 | 0.10 | 0.09 | 0.06 |
| Area 800: Boiler and Turbogenerator |  |  |  |  |
| Boiler chemicals | 0.0023 | 0.01 | 0.0029 | 0.01 |
| Lime | 0.20 | 0.13 | 0.12 | 0.10 |
| Natural gas | − | − | 1.84 | 0.00 |
| Disposal of ash | 0.33 | 0.13 | 0.12 | 0.12 |
| Area 900: Utilities |  |  |  |  |
| Cooling tower chemicals | 0.09 | 0.06 | 0.04 | 0.05 |
| Makeup water | 0.42 | 0.45 | 0.36 | 0.41 |
| Purchased electricity from the grid | − | − | 12.34 | − |
| By-Product Revenue |  |  |  |  |
| Electricity sold to the grid | −2.82 | −3.63 | − | −5.21 |
| **Total Raw Materials Cost (MM $/year)** | **197.6** | **175.3** | **191.8** | **131.7** |
| **Fixed Operating Costs (MM $/year)** | **15.5** | **12.6** | **12.7** | **12.1** |
| **Total Operating Cost (MM $/year)** | **213.1** | **187.9** | **204.5** | **143.8** |

Table S4. Total capital investments of 60 MM gal/year bioethanol plant utilizing two-stage Cu-AHP pretreatments.

| **Process Area** | 30 °C NaOH-H_2_O PE + Cu-AHP  MM $ | 120 °C NaOH-H_2_O PE + Cu-AHP  MM $ | 120 °C NaOH-EtOH PE + Cu-AHP  MM $ | Hypothetical-case as identified by sensitivity analysis  MM $ |
| --- | --- | --- | --- | --- |
| Area 100: Feedstock handling | $25.7 | $27.3 | $26.8 | $27.3 |
| Area 200: Pretreatment | $9.7 | $9.9 | $22.6 | $9.9 |
| Area 300: Enzymatic Hydrolysis and Fermentation | $30.1 | $23.7 | $23.1 | $23.3 |
| Area 400: Enzyme Production | $73.7 | $30.6 | $30.5 | $25.6 |
| Area 500: Recovery | $24.3 | $23.0 | $23.1 | $22.8 |
| Area 600: Wastewater | $51.5 | $51.7 | $48.5 | $50.3 |
| Area 700: Storage | $7.0 | $6.8 | $6.8 | $6.6 |
| Area 800: Boiler/Turbogenerator | $71.3 | $65.3 | $61.3 | $64.5 |
| Area 900: Utilities | $9.8 | $8.0 | $7.1 | $7.3 |
| **Total Installed Costs** | $303.2 | $246.2 | $249.8 | $237.5 |
| Total Direct Costs (TDC) | $327.3 | $261.4 | $267.2 | $251.8 |
| Total Indirect Costs | $196.4 | $156.8 | $160.3 | $151.1 |
| **Fixed Capital Investment (FCI)** | $523.7 | $418.3 | $427.5 | $402.9 |
| Land | $1.8 | $1.8 | $1.8 | $1.8 |
| Working Capital | $26.2 | $20.9 | $21.4 | $20.1 |
| **Total Capital Investment (TCI)** | $551.7 | $441.0 | $450.8 | $424.9 |


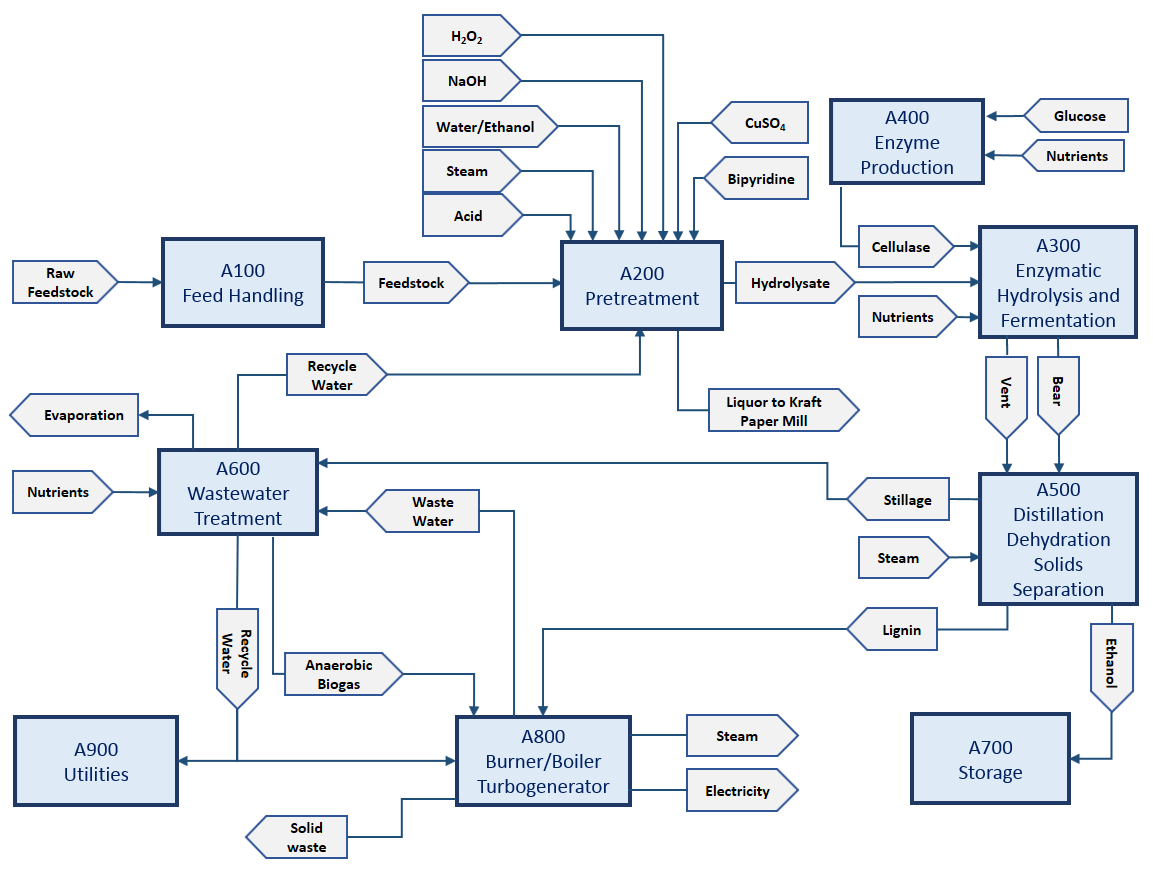


Fig. S1. Block flow diagram of the biorefinery.


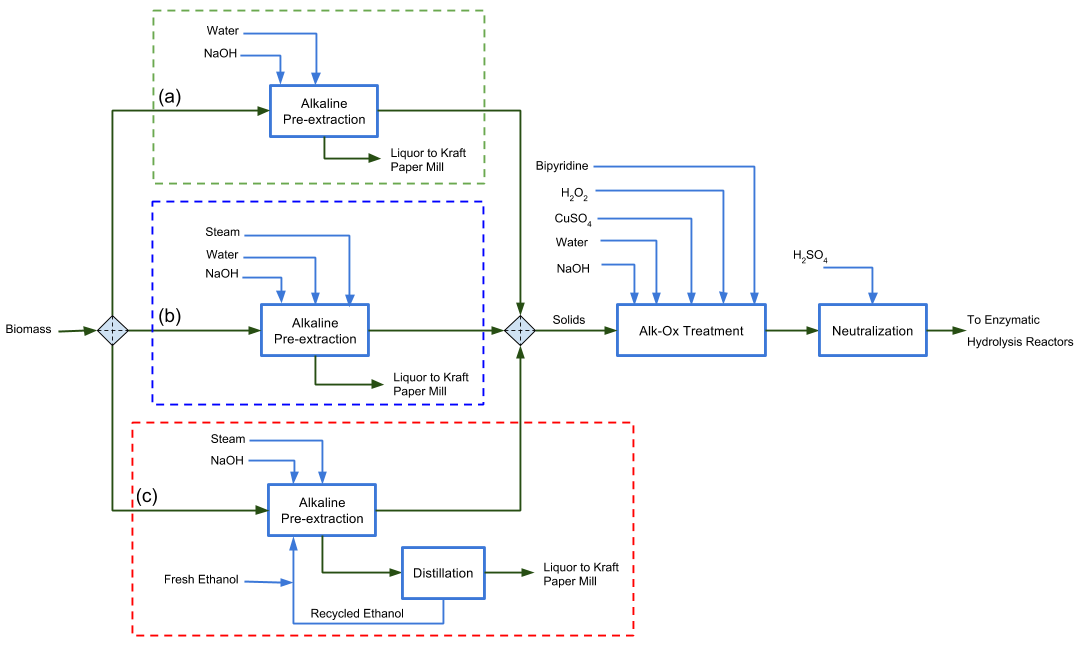


**Fig. S2.** Process flow diagram of pretreatment unit including first stage (a) 30 °C NaOH-H_2_O PE, (b) 120 °C NaOH-H_2_O PE, and (c) 120 °C NaOH-EtOH PE followed by second stage Cu-AHP pretreatment.

**References**

1. Humbird D, Davis R, Tao L, Kinchin C, Hsu D, Aden A, P. Schoen, J. Lukas, B. Olthof, M. Worley, D. Sexton, D. Dudgeon. Process design and economics for biochemical conversion of lignocellulosic biomass to ethanol, Technical Report, NREL/TP-5100-47764, Golden, Co, USA, 2011. <http://www.nrel.gov/biomass/pdfs/47764.pdf>
2. Davis R., Tao L., Tan E.C.D., Biddy M.J., Beckham G.T., and Scarlata C., Jacobson J., Cafferty K., Ross J., Lukas J., Knorr D., Schoen P. Process design and economics for the conversion of lignocellulosic biomass to hydrocarbons: Dilute-acid and enzymatic deconstruction of biomass to sugars and biological conversion of sugars to hydrocarbons, Technical Report, NREL/TP-5100-60223, 2013.
3. Davis R., Tao L., Scarlata C., Tan E.C.D., Ross J., Lukas J., Sexton D. Process design and economics for the conversion of lignocellulosic biomass to hydrocarbons: Dilute-acid and enzymatic deconstruction of biomass to sugars and catalytic conversion of sugars to hydrocarbons, Technical Report, NREL/TP-5100-62498, 2015.
4. Chai L., Saffron C.M., Comparing pelletization and torrefaction depots: Optimization of depot capacity and biomass moisture to determine the minimum production cost. Applied Energy 2016, 163:387–395.
5. Alibaba Group Holding Limited. Alibaba.com. < <https://www.alibaba.com/product-detail/-Trade-Assurance-hydrogen-peroxide-chemical_60205084655.html?s=p>>. [Accessed 2017].
6. Bhalla A., Bansal N., Stoklosa R.J., Fountain M., Ralph J., Hodge D.B., Hegg E.L., Effective alkaline metal-catalyzed oxidative delignification of hybrid poplar. Biotechnol Biofuels 2016, 9:34.
7. Richman Chemical Inc. < http://www.richmanchemical.com/>. [Accessed 2016].
